# Pipette cleaning in automated systems

**DOI:** 10.1155/S1463924686000263

**Published:** 1986

**Authors:** M. L. Severns, J. E. Brennan, L. M. Kline, K. M. Epley

**Affiliations:** Product Development Laboratory American Red Cross 9312 Old Georgetown Road Bethesda Maryland 20814 USA


Journal of Automatic Chemistry/*Journal of Clinical Laboratory Automation*, Vol. 8, No. 3 (July–September 1986), pp. 135–141

### Pipette cleaning in automated systems*

**M. L. Severns, J. E. Brennan, L. M. Kline and K. M. Epley**

*Product Development Laboratory, American Red Cross, 9312 Old Georgetown Road, Bethesda, Maryland 20814, USA*

### Introduction

Recently, a number of robotic sample handling systems have been introduced which perform sample preparation steps in laboratory tests. Many of these systems, such as those described by Severns and Hawk [1] and Martin [2], have sophisticated positioning systems which are under computer control. Some of these robotic systems have been configured as dedicated pipettor/diluters whose positioning system manipulates a rigid tube, or pipette, which is connected to a computer-controlled syringe pump. Systems of this type are quite useful for introducing samples from sample tubes into secondary containers (such as microplates) for performing tests. In addition to minimizing the potential for pipetting errors, these systems can also perform data management functions, such as keeping track of sample identification and test results.

Many of the tests which are performed using automated pipetting systems are not sufficiently sensitive that carry-over between samples or tests introduces significant errors. Certain tests, however, such as the enzyme immunoassays (EIA and ELISA), radioimmunoassay (RIA) and fluorescent immunoassay (FIMA), are sensitive enough that cross-contamination may present a problem. One such assay is the screening procedure for Hepatitis B surface antigen (HBsAg) which is routinely performed on each unit of blood and plasma collected in the United States. Nath and Dodd [3] have reported that the sensitivity of this assay (which is available from various manufacturers as either an ELISA or an RIA) is better than 1 ng of HBsAg per ml of sample, while the highest concentration of HBsAg found in samples is approximately 1 mg per ml. Therefore, cross-contamination from sample to sample must be less than 1 part per million if false positive results due to carry-over are to be avoided. Until recently, this stringent requirement for cross-contamination dictated the use of disposable pipette tips, adding substantially to the cost of assay. It was hoped that a method could be found to clean a non-disposable pipette adequately to perform this test.

One method which is frequently used to clean pipettes in automated systems is to place the tip of the pipette into a 'wash station' and to force liquid through it, as shown in figure 1. The liquid travels through the inside of the pipette, removing contaminants, then up the interior of the wash station, cleaning the outside of the pipette. A substantial advantage of this approach is the low cost of water. The purpose of this study was to determine the primary factors which influence the removal of water-soluble contaminants from a stainless-steel pipette when distilled water is used as the cleaning liquid.

### Methods and equipment

All studies were performed using a Tecan Sampler 505 (Tecan [US] Ltd, Hillsborough, North Carolina, USA). The sampler consists of a Cartesian (X,Y,Z) robotic arm and a pipette connected via Teflon tubing to a pair of motorized syringes. The pipette is a 130 mm long stainless-steel tube of 2 mm outer diameter and 1·4 mm inner diameter. The last 20 mm of its length is narrowed, having an inner diameter of 0·4 mm. The outside of the pipette is coated with Teflon; the inside is not. A capacitance sensor allows the Sampler to detect the surface of any conductive liquid. The entire assemblage is controlled by an IBM Personal Computer using a combination of RatBas [4] and assembly language routines.

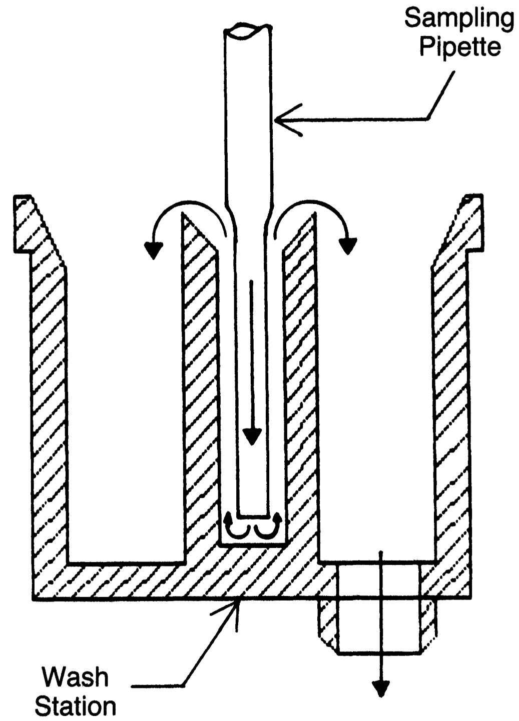

*Figure 1. A schematic drawing of the wash station. Arrows show the direction of fluid flow. Liquid is forced through the pipette, removing contaminants from the inside, then up the sides, removing contaminants from the outside. Liquid exits through the drain in the bottom and is conducted into a collection container.*

* Contribution No. 683 from the American Red Cross.

Two wash stations were investigated. One of them, furnished by Tecan, consisted of a cylindrical Teflon rod with a 3 mm diameter axial hole bored 35 mm into one end. Its internal volume, measured by displacement, was 230 µl. The rod sat inside a cup which allowed liquid to be drained through a 10 mm tube into a waste container. The second wash station consisted of a Teflon rod with a 3 mm axial hole bored 12 mm into one end. Its measured internal volume was 70 µl. Additionally, the top of this wash station was tapered to enhance the removal of liquid.

An initial study, using coomassie blue as an analyte, was performed to determine whether contamination on the pipette was confined to its inside. It was determined that approximately 1·5 µl of liquid adhered to the pipette, partitioned between the outside and the inside surfaces. In the experiments which are described below, the cleanliness of the pipette is estimated by measuring the amount of analyte in liquid which is withdrawn from the wash station. As this liquid was just expelled from the pipette, but has also been in contact with the outside tip of the pipette, it should reflect the likelihood that contamination would be introduced into a subsequent sample.

To determine the effectiveness with which the pipette was being cleaned, the dilution (the ratio of measured concentration to the original concentration) of analyte in the liquid sampled from the wash station was analysed as a function of the volume of distilled water which had been dispensed to clean the pipette. The analytes (all of which were water soluble) were prepared in distilled water, 7% bovine serum albumin (BSA) or human serum. Because of the limited resolution of the measuring instruments, the analytes (except for HBsAg) were prepared in different initial concentrations to extend the range of dilutions which could be measured. The data from a single set of experimental conditions were pooled for analysis.

Several different analytes were used during this study. Coomassie blue (Eastman Kodak Company, Rochester, New York, USA) concentrations were determined using a Dynatech MR600 microplate reader (Dynatech Laboratories, Inc., Alexandria, Virginia, USA) at a wavelength of 630 nm. The concentration of fluorescein (sodium salt, Sigma Chemical Co., St. Louis, Missouri, USA) was determined using a Turner model 112 filter fluorometer with a microcuvette adapter (Sequoia-Turner Corporation, Mountain View, California, USA). 4-methylumbilleferone (MUB, Sigma Chemical Co., St. Louis, Missouri, USA) was prepared in solution at pH 11. Its concentration was determined using a Dynatech MicroFluor Autoreader (Dynatech Laboratories, Inc., Alexandria, Virginia, USA). Sera containing Hepatitis B surface antigen (HBsAg) were obtained from the American Red Cross Transmissible Disease Laboratory. Dilutions of HBsAg were determined using an ELISA technique (Auszyme II, Abbott Laboratories, Chicago, Illinois, USA).

In all of the studies, except those utilizing HBsAg, each experiment was calibrated to determine the relationship between the measured variable (absorbance or fluorescence) and the dilution; 24 serial two-fold dilutions were prepared by the Sampler. A technologist later added distilled water to each well so that the total volume was the same as in the actual wash procedures (250 µl). The calibration thus consisted of dilutions from 2 : 1 to 16 777 216 : 1.

Each experiment was conducted on the same microplate as its calibration curve. For each wash volume, an air gap of 7 µl (to minimize mixing of the analyte with the wash solution) was first aspirated into the pipette. The Sampler then moved the pipette to the surface of the analyte (as determined by the liquid level sensor) and aspirated an aliquot which varied in volume depending upon the experiment. The Sampler was programmed to follow the surface of the liquid, remaining just sufficiently submerged (less than 1 mm) to avoid aspirating air. The pipette was moved to the wash station, where the analyte and air gap were dispensed, followed by the proper volume of wash solution. A 50 µl aliquot of liquid from the wash station was aspirated and placed into a well of the microplate along with 200 µl of wash solution (to bring the liquid level sufficiently high that no errors were introduced in the reading process). Finally, the pipette was returned to the wash station where an additional 2000 µl of wash solution was dispensed.

### *Data analysis*

Calibration and experimental data were entered into a VAX-11/780 computer (Digital Equipment Corporation, Marlborough, Massachusetts, USA) for analysis. The majority of analysis was performed using the RS/1 (BBN Software Products, Cambridge, Massachusetts, USA) and BMDP (University of California at Los Angeles) software packages.

Because of changes in such items as protein concentration (when BSA or plasma was used as the carrier for the analyte) and pH (when MUB was used as the analyte) with dilution, there was often a nonlinear relationship between the measured variable (especially fluorescence) and dilution. To account for some of this variability, a second order polynomial was fitted to the calibration data using the method described by Forsythe [5]. The coefficients of the polynomial were used to compute the dilutions from the experimental measurements.

After the experimental dilutions had been calculated, all data from a single set of experimental conditions were pooled. A minimum least squares fit of the appropriate model equation to the pooled data was determined using the Marquardt–Levenberg technique [6] or a pseudo Gauss–Newton technique described by Dixon [7]. As the expected form of the washout curves was a sum of exponentials, model equations were fit to the logarithm of the data points, so that all data points would influence the fit equally.

To determine which of two model equations best fit the data, the residuals from both models were examined to determine which explained a greater portion of the total variance. Residuals were first examined to see if they were normally distributed using the Wilk–Shapiro test. If both sets of residuals were normally distributed, their variances were tested using the F-test, otherwise they were tested using the Ansari–Bradley test. The application of these tests is described by Sokal and Rohlf [8].

To test data from two experimental conditions for equality, a two-way analysis of variance (ANOVA) [8] was performed on the data from the washout curves using experimental condition and wash volume as the classification variables. Parameter values derived from curve fitting were tested for equality using Student's t-test. Standard error estimates from the model fit were used to approximate the standard deviation of the estimate, and the number of degrees of freedom was computed as $N_1 + N_2 - 2$, where $N_1$ and $N_2$ are the degrees of freedom from the parameter estimates.

### Results

*Effect of depth of submersion in the wash station*

Figure 2(*a*) shows the relationship between dilution and wash volume in the large wash station when the pipette is immersed 5 mm (close to the surface). The analyte used was coomassie blue. A one-compartment model (Equation 1, Appendix A) explained significantly less of the variability in the data than did a cascaded two-compartment model (Equation 2, Appendix A, $p = 0{\cdot}005$, Ansari–Bradley test). In this case, the two compartments of the model appear to represent portions of the wash station where liquid does not mix well.

Figure 2(*b*) shows the relationship between dilution and wash volume in the large wash station when the pipette is immersed 25 mm (close to the bottom). In this case, the two compartment model does not yield a better fit to the data than the one compartment model ($p = 0{\cdot}88$, F-test). Additional experiments at various submersion depths indicate that the one compartment model is appropriate when the pipette is inserted at least 15 mm into the wash station cavity.

It requires significantly more liquid to attain a specified dilution when the tip of the pipette is submerged 5 mm than when it is submerged 25 mm ($p < 0{\cdot}0001$, two-way ANOVA). In addition, the estimated compartmental volume is decreased from 234 µl ($V_1$ = 119 µl, $V_2$ = 115 µl) to 87 µl ($V_1$ = 87 µl). Thus, the pipette is cleaned in a more satisfactory fashion when its tip is placed near the bottom of the wash station. In the remainder of the experiments described, the tip of the pipette is placed 5 mm from the bottom of the wash station.

*Effect of depth of wash station cavity*

The relationship between wash volume and dilution of analyte was determined in both of the wash stations. The larger wash station had a measured interior volume of 230 µl, of which the pipette displaced approximately 37 µl. The smaller wash station had a measured interior volume of 70 µl, of which the pipette displaced approximately 12 µl. Coomassie blue was used as the analyte and both washout curves were fit using Equation 2 (Appendix A). Despite a three-fold difference in the internal volumes

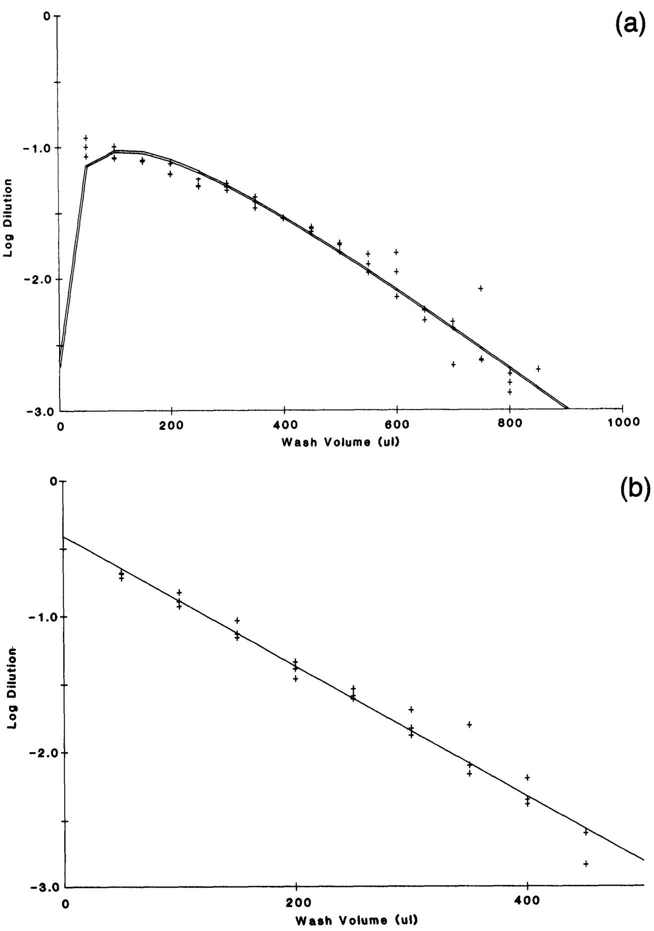

*Figure 2 (a). Washout of coomassie blue from the large wash station with the pipette submerged 5 mm (close to the surface). Line through data points is best fit of Equation 2, Appendix A (A = 8·03; $V_1$ = 118·9; $V_2$ = 115·1). (b) Washout of coomassie blue from the large wash station with the pipette submerged 25 mm (close to the bottom). Line through data points is best fit of Equation 1, Appendix A (A = 0·39; $V_1$ = 90·6).*

of the wash stations, the differences in the estimated values of $V_1$ were not statistically significant ($p = 0{\cdot}17$, two-way ANOVA). The estimate of the initial concentration was significantly lower in the smaller wash station ($p < 0{\cdot}001$, two-way ANOVA). Thus, the depth of the wash station appears to have a small but definite effect on the washout of contaminants near the tip of the pipette. Because both wash chambers had the same inside diameter, it was not possible to examine the effect of cross-sectional area.

*Effect of volume of sample*

When a more sensitive analyte is used to study the washout process, it becomes apparent that the curve changes slope at high dilution. This change seems to reflect a second process which dominates the washout process for dilutions greater than approximately 1000 : 1. Figure 3 shows typical experimental data generated using MUB as an analyte. The curve was fit using Equation 3 (Appendix A).

The parameter estimates values for the fast portion of the washout (A and $V_1$) are unaffected by the volume of

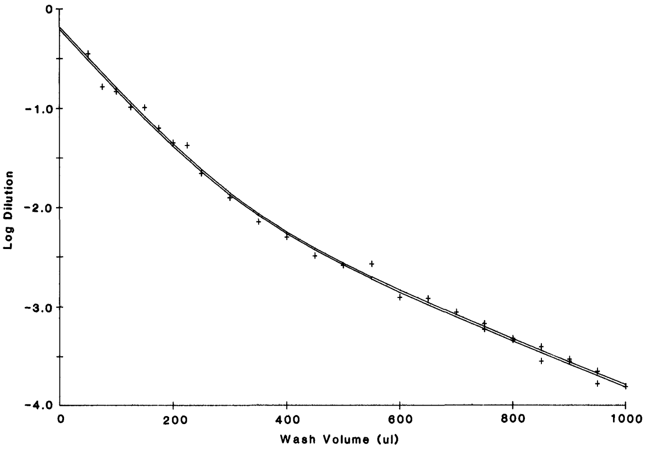

*Figure 3. Washout of 4-methyl umbelliferone from the small wash station. Line through data points is best fit of Equation 3, Appendix A ($A = 0{\cdot}61$; $V_1 = 67{\cdot}3$; $B = 0{\cdot}033$; $V_2 = 187{\cdot}1$).*

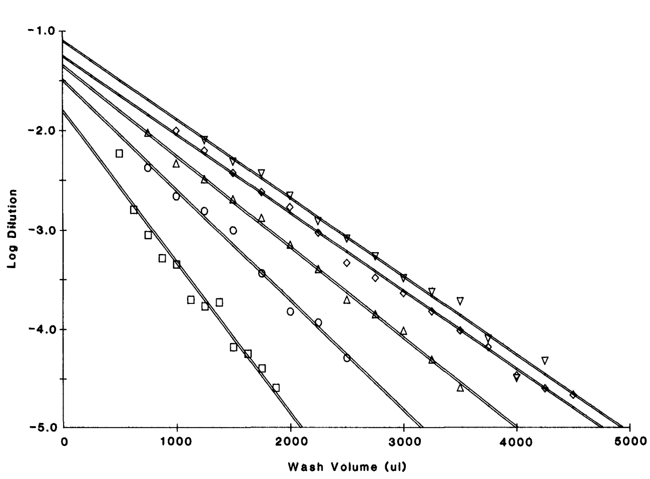

*Figure 4. Washout of analyte from the pipette when different initial volumes of analyte were aspirated. Volumes aspirated were 50 µl (□), 100 µl (○), 150 µl (△), 200 µl (◇) and 250 µl (▽). Lines through the points were best fits of a single exponential decay function.*

analyte which was aspirated ($p > 0{\cdot}38$, t-test following linear regression on the differences). The parameter estimates for the slow portion of the washout ($B$ and $V_2$) are both strongly dependent upon the volume of sample aspirated ($P < 0{\cdot}001$, linear regression on parameter values). Washout curves for various sample volumes are shown in figure 4.

*Effect of changing the wettability of the pipette surface*

To determine if the wettability of the surface of the stainless-steel pipette affected the removal of contaminants, a stainless-steel pipette was exposed for 12 h to a 1% solution of Prosil-28 (PCR Research Chemicals, Inc., Gainesville, Florida, USA), an organosilane surface treating compound, then air dried at 75 °C for 1 h. This treatment was found to substantially reduce the contact angle [9] of water droplets on stainless-steel, indicating that the surface had become considerably more hydrophobic. No difference was found in the washout of MUB between treated and untreated pipette ($p > 0{\cdot}5$, two-way ANOVA).

To further verify the lack of effect of surface wettability, Tween-20 (polyethyleneoxidesorbitan laurate, Sigma Chemical Co., St. Louis, Missouri, USA), a surface wetting agent, was added to the distilled water which was flushed through the pipette. No change in the washout of MUB was detected ($p > 0{\cdot}7$, two-way ANOVA).

*Effect of the viscosity of the solution containing analyte*

To determine if the viscosity of the solution containing the analyte affected the washout process (as it might if analyte is trapped in a film at the wall), fluorescein was used as an analyte in both distilled water (viscosity = 1 centipoise) and in 7% BSA (viscosity = 1·8 centipoise). No difference was found in the washout curves for these two experimental conditions ($p > 0{\cdot}5$, two-way ANOVA).

*Effect of diffusion of analyte*

If diffusion of the analyte effects the washout from the pipette, as in Taylor dispersion [10], then two effects should be evident. First, if the diffusion coefficient of the analyte decreases, the estimate of the value of $V_2$ (Equation 3, Appendix A) should increase. Additionally, a decrease in the flow rate should cause a decrease in the estimate of the value of $V_2$ (Equation 3, Appendix A).

To determine if the flow rate had an effect on washout, fluorescein was used as an analyte in experiments at two different flow rates; 2·6 cc/s (the highest flow rate which was possible without overloading the syringe pump motor) and 0·5 cc/s. The calculated Reynold's number in the 1·4 mm wide portion of the pipette is 3310 at the higher flow rate, and 637 at the lower one (the critical Reynold's number for turbulent flow is 2300). In the tip, which has a narrower inside diameter, the Reynold's numbers rise to 20690 and 3980, respectively. A small but statistically significant ($p < 0{\cdot}0005$, t-test on parameters) change in the estimate of $V_2$ was found, from 250 µl at the high flow to 313 µl at the lower one. This change is in the opposite direction from that which would be expected if diffusion were an important factor in the washout process.

To determine the effect of diffusion on the washout process, analytes of different molecular weights were used. Studies were performed using MUB (molecular weight 198·2) and HBsAg (approximate molecular weight $2{\cdot}7 \times 10^6$). Table 1 shows the estimates of $V_2$ for these conditions; the estimates are significantly different ($p < 0{\cdot}001$, t-test on parameters). It appears that the molecular weight of the analyte has a small but detectable effect on the washout process. Since the coefficient of diffusion of a substance is (to a first approximation)

*Table 1. Estimates of slope of washout curve for analytes of different molecular weights. Sample volume was 200 µl.*

| Analyte | Molecular weight | Estimate of $V_2$ |
|---|---|---|
| MUB | 198·2 | 553·0 |
| HBsAg | $2{\cdot}7 \times 10^6$ | 1072·0 |

related to the inverse of the square root of the molecular weight, the ratio of the diffusion coefficients of the two analytes is on the order of 100 : 1. This change in diffusion coefficient resulted in a 2 : 1 change in the estimated value for $V_2$.

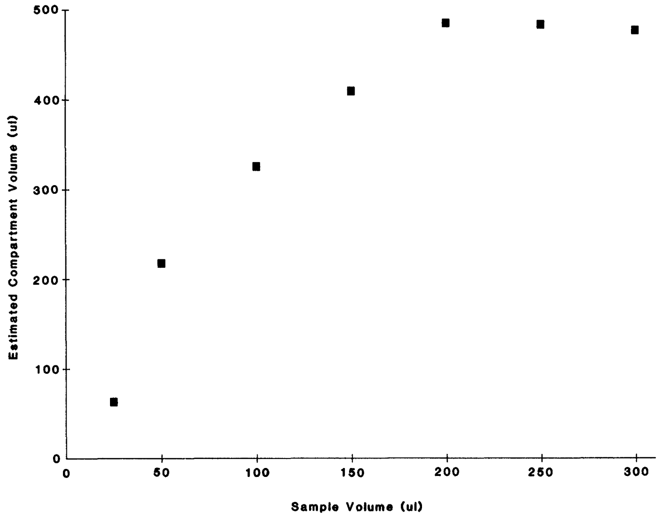

*Figure 5. Relationship between the volume of sample aspirated and the parameter $V_2$ in Equation 3, Appendix A.*

### Conclusions

The washout of contaminants from a pipette in a wash station (assuming that the tip of the pipette is close to the bottom of the wash station) can be modeled by two parallel well-stirred compartments. The response of the system consists of an initial (fast) portion and a later (slow) portion.

The initial part of the washout process appears to be related to the convective transport of analyte in the wash station itself. It is relatively unaffected by the amount of sample aspirated or by the diffusivity of the analyte; it is slightly affected by the depth of the wash station. The initial portion of the washout process appears to be most affected by the depth to which the pipette is inserted into the station. When the pipette is inserted only a small distance into the wash column, the flow in the column appears to be partitioned into at least two separate 'compartments', which do not mix well. It is possible that the partitioning reflects the existence of a plume of liquid. When the pipette is lowered sufficiently into the wash column, the increased turbulence and decreased volume appear to cause better mixing.

The latter part of the washout process appears to be primarily related to the removal of analyte from the inside of the pipette. This portion of the washout process is significantly affected by the amount of sample which is aspirated and, to a lesser extent, by the flow rate and the diffusion coefficient of the analyte. When the flow rate was changed, the slope of the curve changed in a direction opposite to that predicted if diffusion were a dominant force in the washout process. It therefore appears that the washout of analyte during the second phase of the curve is primarily due to convective transport, and only

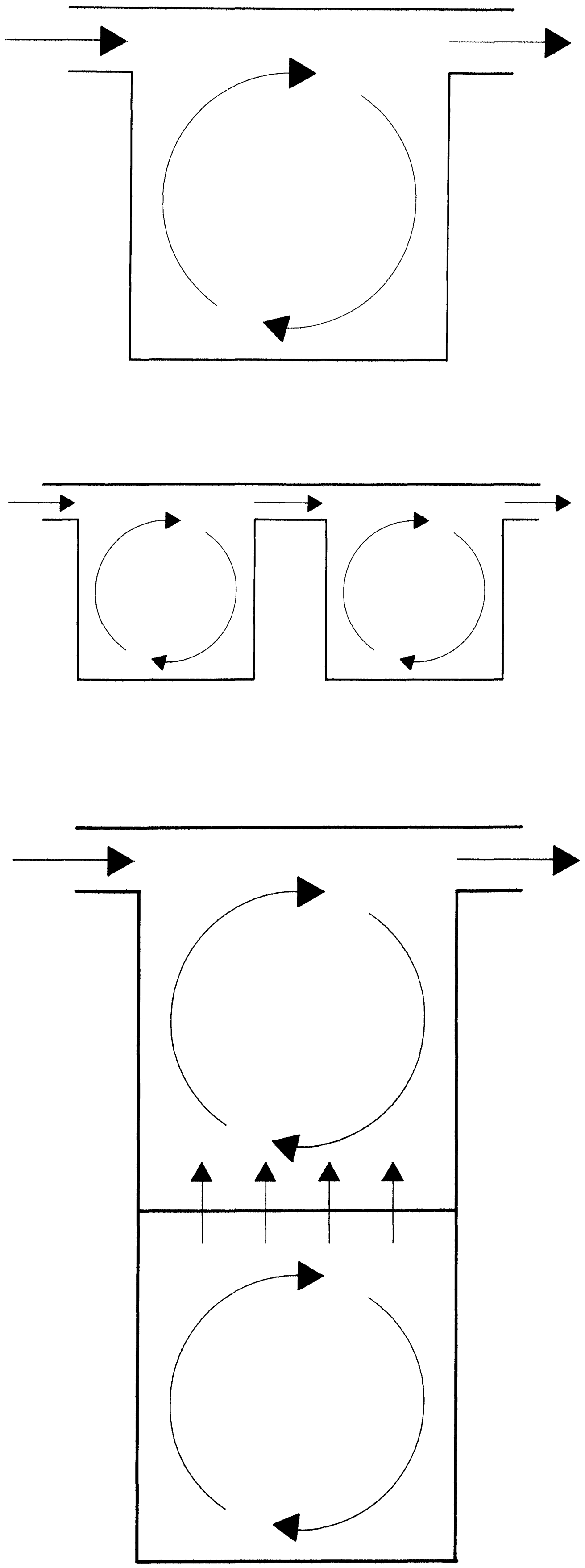

*Figure 6. Various models which were used to describe the washout process. (a) One compartment model. (b) Two cascaded compartment model. (c) Two parallel compartment model.*

secondarily due to diffusion of analyte. It is possible that the apparent effect of the molecular weight of the analyte is due to a non-diffusional binding effect at the wall. Further experiments with analytes of varying molecular weights may determine the nature of this effect.

Attempts were made to explain the washout of analyte using existing models of the dispersion of solute in a tube (Equations 4, 5 and 6, Appendix A). None of these models improve the explanation of the data, and the models for laminar flow with no diffusion (Equation 4), and turbulent flow (Equation 6) are significantly worse ($p < 0{\cdot}001$, F-test).

The dependence of estimates of $V_2$ on the volume of sample aspirated is striking. Figure 5 shows the relationship between the volume of sample aspirated and the estimate for the parameter $V_2$ (Equation 3, Appendix A). Since the measured interior volume of the pipette was 200 μl, the break which is visible in the curve may be due to the transition from the stainless-steel pipette to the Teflon connecting tubing. It appears that the change in slope may therefore be related to a change in the exposed surface area.

To determine if the predictions of the model system could be validated in practice, a pseudo-random sequence of 30 HBsAg positive samples and 30 negative controls were pipetted into an Abbott tray for the Auszyme II assay by the Tecan Sampler. A volume of 7 ml (predicted from the experiments above to adequately reduce carry-over) was used to wash the pipette between samples. No trace of carry-over was found in any of the negative samples.

The results of this study have been utilized by Brennan *et al.* [11] in an automated system which transfers samples from tubes of donor blood into trays to perform HBsAg and anti HTLV-III assays. This system has been used to test over 10 000 samples without evidence of sample cross-contamination.

### Acknowledgements

We would like to thank Ms Rebecca Akins for performing the HBsAg assays, and Dr L. I. Friedman for reviewing several drafts of this manuscript.

### References

1. Severns, M. L. and Hawk, G. L., In *Robotics and Artificial Intelligence (NATO ASI Series F, Volume 11)*, Ed. Brady, M., Gerhardt, L. A. and Davidson, H. F. (Springer-Verlag, Berlin, 1984), p. 633.
2. Martin, W., *American Laboratory*, **17** (1985), 178.
3. Nath, N. and Dodd, R. Y., *CRC Handbook Series in Clinical Laboratory Science, Section D: Blood Banking, Volume 2.* Ed. Greenwalt, T. J. and Steane, E. A. (Chemical Rubber Co., Boca Raton, 1981), p. 301.
4. Sharpe, W. F. and Weaver, B., *RatBas—a software tool for users of BASIC* (Wells Fargo Investment Advisors, San Francisco, 1982).
5. Forsythe, G. E., *Journal of the Society for Industrial and Applied Mathematics*, **5** (1967), 74.
6. Marquardt, D. W., *Journal of the Society for Industrial and Applied Mathematics*, **11** (1963), 431.
7. Dixon, W. J., *BMDP Statistical Software* (University of California Press, Los Angeles, 1983), p. 674.
8. Sokal, R. R. and Rohlf, F. J., *Biometry* (Freeman, San Francisco, 1981).
9. Good, R. J., In *Surface and Colloid Science.* Ed. Good, R. J. and Stromberg, R. R. (Plenum Press, New York, 1979), p. 1.
10. Taylor, G., *Proceedings of the Royal Society A*, **219** (1953), 186.
11. Brennan, J. E., Severns, M. L. and Kline, L. M., In *Advances in Laboratory Automation Robotics, Volume 2.* Ed. Strimaitis, J. R. and Hawk, G. L. (Zymark Corporation, Hopkinton, 1986) p. 481.
12. Cobelli, C. and Romanin-Jacur, G., *Mathematical Biosciences*, **30** (1976), 139.
13. Severns, M. L. and Adams, J. M., *Journal of Theoretical Biology*, **97** (1982), 239.
14. Taylor, G., *Proceedings of the Royal Society A*, **223** (1954), 446.
15. Aris, R., *Proceedings of the Royal Society A*, **252** (1959), 538.

### Appendix A

*Models of the washout process*

A number of standard models have been used to analyse data from the pipette cleaning experiments. The assumptions involved in formulating each of the models, and the equations which result, are briefly reviewed here in the context of the washout process. A more detailed treatment of the compartmental models is presented by Cobelli and Romanin-Jacur [12].

The simplest model which can be used to describe the washout process is the 'well-stirred compartment' model. In this model, liquid entering a compartment of fixed volume is assumed to mix instantaneously with the fluid in the compartment. The concentration of analyte in the fluid leaving the compartment is the same as the concentration of analyte in the compartment. This process is shown schematically in Figure 6($a$).

Let the dilution of analyte D be given by $C/C_0$, where $C$ is the time varying analyte concentration and $C_0$ is the concentration of analyte injected into the system at time $t = 0$. If the concentration of analyte in the incoming liquid is zero, then the relationship between wash volume and dilution is:

$$\mathrm{D} = A\, e^{-W/V_1} \qquad (1)$$

where $W$ is the volume of liquid which has passed through the compartment since time $t = 0$, $V_1$ is the volume of the compartment and $A$ is the ratio of the concentration of analyte in the compartment at time $t = 0$ to the concentration of analyte injected.

A cascade of two well-stirred compartments is shown in figure 6($b$). In this model, liquid enters the first compartment, mixes completely, and then flows into the second compartment. Liquid mixes completely in the second compartment before exiting the system. This model is used to fit the data which resulted from experiments where the tip of the pipette was located close to the top of the column of liquid in the wash station. In this case, the two 'compartments' appear to represent portions of the liquid in the wash station which mix poorly. If the initial concentration in the first and second compartments at time $t = 0$ are $C_0$ and 0, respectively, and the concentration of analyte in the liquid entering the first compartment is zero, then the resulting equation for the output of this model is given by:

$$D = A(e^{-W/V_1} - e^{-W/V_2}) \quad (2)$$

where $V_1$ is the volume of the larger of the cascaded compartments, $V_2$ is the volume of the smaller compartment and A is a constant which is related to the rate of flow of wash solution and to the compartmental volumes.

A model which appears to fit much of the experimental data is the two parallel compartments model. The analyte contained in one compartment, which is isolated from the flow of liquid, is transported into the second compartment, through which liquid flows. This model is shown schematically in Figure 6(*c*). Severns and Adams [13] provide a detailed discussion of the assumptions which are involved for geometries similar to those of a thin film on the inside of a tube. The equation which describes the output of this model is:

$$D = A\,e^{-W/V_1} + B\,e^{-W/V_2} \quad (3)$$

where $V_1$ and $V_2$ are a function of the volumes of the compartments, the rate of flow and the transport coefficient between compartments, and $A$ and $B$ are dependent upon the initial conditions in the compartments.

Taylor [10 and 14] and Aris [15] have described models to predict the dispersion of analyte in liquid flowing through a tube. The form of the dispersion of analyte depends upon whether the flow is laminar or turbulent and on the relative magnitudes of convective transport and diffusional transport of analyte. If the flow is laminar and transport of solute by diffusion negligible in comparison to convective transport, then the equations which describes the process are:

$$\begin{aligned} D &= B(1 - W/A) & W/A < 1 \\ D &= 0 & W/A > 1 \end{aligned} \quad (4)$$

where $B$ is the ratio of the concentration of analyte in the test solution to the concentration of analyte in the compartment at time $t = 0$ and $A$ is the cross-sectional area of the tube. If diffusion across the radius of the tube occurs in a time similar to the characteristic time for axial convective transport, ana if the flow is constant (so that the wash volume is directly proportional to the time), then the equation describing the process is:

$$\begin{aligned} D &= A \left\{1 - erf\left[\frac{W - W_0}{B\sqrt{W}}\right]\right\} & W < W_0 \quad (5) \\ D &= A \quad 1 + erf\ \frac{W - W_0}{B\sqrt{W}} & W > W_0 \end{aligned}$$

where $erf(x)$ is the error function, given by

$$erf(x) = \frac{2}{\pi} \int_0^x e^{-x^2}\, dx$$

the constant $A$ is related to the initial dilution of analyte, and $B$ is related to the rate of flow through the tube and the diffusivity of the analyte. If the flow is constant and of sufficient magnitude that turbulence occurs, the equation which describes the process is:

$$D = A\, W^{\frac{1}{2}}\, e^{-(W - W_0)^2/BW} \quad (6)$$

where $A$ and $B$ are constants related to the dispersion of the solute in the tube and $W_0$ is the amount of liquid necessary to transport 1/2 of the analyte from the initial point to the measuring point.

**SAC 86/3rd BNASS:**
**An International Conference on Analytical Chemistry and Atomic Spectroscopy**

This conference is being organized by the Analytical Division of the Royal Society of Chemistry, in conjunction with the Spectroscopy Group of the Institute of Physics, and will be held at the University of Bristol from Sunday to Saturday, 20 July to 26 July 1986.

*Application forms are now available and may be obtained from: Miss P. E. Hutchinson, Analytical Division, Royal Society of Chemistry, Burlington House, London W1V 0BN. Tel: 01 437 8656.*

